# Serum Oxytocin in Cows Is Positively Correlated with Caregiver Interactions in the Impossible Task Paradigm

**DOI:** 10.3390/ani12030276

**Published:** 2022-01-23

**Authors:** Biagio D’Aniello, Vincenzo Mastellone, Claudia Pinelli, Anna Scandurra, Nadia Musco, Raffaella Tudisco, Maria Elena Pero, Federico Infascelli, Alfredo Di Lucrezia, Pietro Lombardi

**Affiliations:** 1 Department of Biology, University of Naples Federico II, 80126 Naples, Italy; anna.scandurra@unina.it (A.S.); a.dilucrezia@hotmail.it (A.D.L.); 2 Department of Veterinary Medicine and Animal Production, University of Naples Federico II, 80137 Naples, Italy; vincenzo.mastellone@unina.it (V.M.); nadia.musco@unina.it (N.M.); tudisco@unina.it (R.T.); mp3054@cumc.columbia.edu (M.E.P.); infascel@unina.it (F.I.); pilombar@unina.it (P.L.); 3 Department of Environmental, Biological and Pharmaceutical Sciences and Technologies, University of Campania “Luigi Vanvitelli”, 81100 Caserta, Italy; claudia.pinelli@unicampania.it; 4 Department of Pathology, Anatomy and Cell Biology, Columbia University, New York, NY 10032, USA

**Keywords:** behavior, cortisol, dairy cows, human–animal interaction, impossible task, oxytocin

## Abstract

**Simple Summary:**

A possible relationship between circulating hormones (e.g., oxytocin and cortisol) and social behaviors toward humans in cows was studied using the impossible task paradigm test. Serum oxytocin levels correlated positively with the duration and negatively correlated with the latency of the cows’ social interactions with the caregiver. The implications of these findings for productivity as well as for animal welfare can be numerous and worth further investigation.

**Abstract:**

This study explored a possible relationship between the circulating oxytocin, cortisol, and the willingness of dairy cows to engage in social behaviors with humans in an experimental context. The behaviors of twenty-nine cows were recorded during the impossible task paradigm, a procedure aimed at creating a violation of expectancy, in the presence of the caregiver and a stranger. The results showed that serum oxytocin levels were positively correlated with duration and negatively correlated with the latency of the cows’ social interactions with the caregiver. This research provides a clear correlation between circulating oxytocin and a willingness to engage in social contact with the caregiver, excluding the possible effect of different cortisol levels on such behavior.

## 1. Introduction

The active nonapeptide oxytocin (OXT) was originally identified as a female hormone due to its important role during delivery and lactation in mammals [1], in egg laying of oviparous vertebrates [2], and in facilitating the mother–infant bond in species showing parental care [3,4]. Currently, it has been established that OXT also serves several other behavioral and physiological functions in both sexes [5,6]. A body of research has focused on the effects of OXT on sociability [7,8], showing its involvement in determining and regulating some social behaviors. In the prairie vole (*Microtus ochrogaster*), OXT favors monogamous relationships with specific partners in females, while OXT antagonists decrease such preference [9]. Furthermore, OXT promotes social bonds by acting on social memory to allow recognizing mates in mice [4,10] and inducing alloparenting behaviors [11]. Several studies have also assessed important roles of OXT in livestock-domesticated species. In sheep, OXT levels are correlated with offspring acceptance after lambing and the mother–offspring bond during lactation [12,13,14] and with social affiliative behaviors in cattle [15]. It has also been suggested that postnatal OXT may have an organizational effect on the brain, as calves with higher plasma OXT levels in early life express higher social behaviors in late life, including both attachment and affiliative behaviors between peers [16]. Moreover, exogenous OXT has been shown to favor affiliation and approach behaviors between dogs as pets [17].

Besides intraspecific studies, the involvement of OXT has also been highlighted in the interspecific relationships. In the last decades, man’s best friend has become the “scholar’s best friend” [18], thus, unsurprisingly, most research has highlighted OXT’s contribution in promoting the special bond between dogs and humans [19]. Particularly, it has been proven that the baseline levels of OXT were positively correlated with social gazing toward humans [19,20]. Moreover, intranasal OXT applications have enhanced the social bonding of dogs with the owner [17], the dogs’ performance in following human cueing [21,22] and the willingness to rescue the owners when pretending to be trapped and stressed [23]. Overall, these last studies provide support for similar social effects of OXT in both intraspecific and interspecific social relations and suggest that OXT could have been an important keystone for the domestication process in dogs [2]. Intuitively, cattle underwent a different domestication process than dogs, having been domesticated for different reasons, further than having a different ecology [24]. Thus, assessing whether OXT could modulate social behaviors between humans and cattle is of particular interest. To our knowledge, inquires on the social effects of blood OXT levels in cattle have been performed in intraspecific studies [15,16], while very few attempts have been made to explore interspecific relationships with humans [25], which is the topic we addressed in the current study.

One of the tools for studying the tendency of animals to perform interspecific social interactions with humans is the impossible task paradigm (IT) [26]. This experimental paradigm consists of some solvable trials in which the subject learns how to obtain a reward from an apparatus in an easy way, followed by an unsolvable phase through the blocking of the apparatus. This procedure triggers a violation of expectation, which forces the animal to change its strategy in an attempt to solve the task, increasing social contacts. Therefore, the IT is considered a useful tool for studying the decision-making process of the animals and their tendency to socially relate to humans (see [27]). Consistent with this goal, the IT has been applied in several canid species (see [28] for a review), but it has also been employed in other domesticated ungulates (horses:. [29]; goats:. [30,31,32]), and in kangaroos [33]. As OXT affects social decision-making mechanisms in humans [34], the IT appears particularly appropriate for studying the effects of OXT in animal–human interactions. Indeed, the IT has already been used in dogs to show that intranasal administration of OXT increases social contact with humans [35]. The IT was also utilized by Kovács et al. [20], who demonstrated that OXT can elicit opposite responses according to the dog’s breed (e.g., while Border Collies increased their gazing behavior towards a stranger, Siberian Huskies reduced this behavior after OXT intranasal injection). Some studies highlighted a link between OXT and stress. Increased stress responses were recorded in mice lacking the OXT gene during maze exploration [36] and a similar result was observed after intranasal OXT administration during a social isolation test in ewes [37]. OXT mediates the stress responses in the presence of social partners (social buffering) [38]; also, synthetic OXT administration helps to normalize the emotional state from a stressful event, favoring the recovery of normal behaviors [39]. It has been hypothesized that OXT may cause endocrine changes in the activity of the hypothalamus–pituitary–adrenal axis by acting as a key modulator of anxiety-related behaviors (reviewed in [40]) and several studies have reported the involvement of OXT in the control of social stress by reducing the cortisol response [41,42,43,44]. Conversely, OXT did not seem to be involved in social anxiety in dogs [17] and, in cows, it specifically enhanced social motivation rather than reducing anxiety and fearfulness for social contexts [16]. Furthermore, centrally administered OXT reduced cortisol secretion in Holstein steers [45].

In the current study, we applied the IT to explore possible relationships between the level of circulating OXT and the willingness of dairy cows to engage in social behaviors with humans. Considering the relationships between OXT, cortisol and social stress, we included the cortisol assay to search for any possible link between OXT and social contacts. In addition to the caregiver, we also included a stranger in the testing procedure to reveal possible familiarity-linked behavioral responses towards people. Most of the previous studies on domestic animals have not found different behavioral outcomes between caregivers and strangers in the IT (horses: [29]; pet dogs: [46,47,48]), with the exception of a single paper on pet dogs, in which a lower latency to gaze the owner was reported [49]. Importantly, owner relevance in dogs can be enhanced by specific training, such as agility [46] and water rescue [47], thus suggesting that the skill to select the familiar person as the main reference point is quite flexible. On the other hand, as the relationship between caregivers and cows is quite different from dogs, it is important to explore possible differences in the social interaction of cows toward familiar and unfamiliar people, testing the correlation with OXT and cortisol according to familiarity.

Based on the cited literature, we expected a positive relationship between the level of circulating OXT and the tendency of cows in attempting social contact with the caregiver. In addition, while OXT increased positive responses towards familiar people, an opposite effect towards strangers was noted (human: [50]; dog: [35]), whereby we should also find a negative correlation between OXT and social contacts with the stranger. The conflicting results on the relationships between OXT and cortisol do not allow for a prevision about cortisol. Likewise, inconsistent data relating to owner discrimination did not allow predicting whether the cows should have shown social preferences according to familiarity.

## 2. Materials and Methods

### 2.1. Animals

The study was carried out on Italian Red Pied (*n* = 29), with an average age of 5.47 ± 2.02 years (min = 2.90, max = 11.30), days in milk 90 ± 15, in a farm located in a hilly area of South Italy (Roccabascerana, Avellino, Italy; longitude 14°42 E, latitude 41°03 N, altitude 380 m above sea level). The animals were housed in a single stable with outdoor access to a 10 ha pasture. The indoor space was 12 m Mori /head, with automatic water bowls. The feed was provided indoors ensuring a linear space of 75 cm/head; in addition, the cows had free access to the outdoors throughout the whole day. Besides the free pasture, the diet ingredients (kg as fed) were mixed hay (*Vicia sativa*, *Hedysarum coronarium*, *Avena sativa*, *Lolium multiflorum*, *Trifolium alexandrinum*) 2.0, alfalfa hay 2.5, cornmeal 2.0, triticale 1.0, and faba bean 2.0. On the farm, a caregiver has been caring for the cows for more than 5 years, a period enough long to consider him familiar. He provides the cows with basic daily care: guides and assists them during milking, feeds them, releases them in the morning, and brings them back to the stable at sunset, cleans them, and carries out the first check on their health.

Ethical clearance was provided by the Ethical Animal Care and Use Committee of the University of Naples Federico II based on the Italian Legislative Decree 26/2014 (art. 2).

### 2.2. Testing Procedure

To induce the expectancy violation, an openable experimental apparatus was constructed by fixing the lid of a transparent plastic food container upside down (diameter 30 cm) on a wooden platform (60 × 40 cm) with 5 screws. The container was loosely positioned upside down on the lid during the solvable trials and was locked during the unsolvable ones. Three people were involved in the test: two experimenters (unknown to the cows) and the caregiver. One experimenter played the role of the stranger; another experimenter manipulated the apparatus and the feed during the procedure and moved away from the experimental area during the trials. Immediately after automatic milking, the cows reached the feeding pen equipped with the apparatus and a Sony’s (Tokyo, Japan) HDR-PJ260VE camera. During the experiment, access to the testing area was denied to other cows or people to avoid interfering with the behavioral repertoire of the testing cow. The apparatus was placed in front of the cow’s feeding pen, while two motionless people were positioned on either side of the apparatus: the caregiver was chosen as a familiar person, and an experimenter assumed the role of the stranger. The position of the caregiver and the stranger (e.g., to the left or right of the cow) was balanced between tests. The cow could reach the apparatus and people with its muzzle by placing its head between the grids of the pen, as when they regularly receive feed.

The training procedure consisted of three consecutive solvable trials, followed by the test in the unsolvable trial. In the training phase, the experimenter put a palatable feed (cornmeal) on the lid fixed and turned upside down on the wooden platform; the cow could reach it by moving the container sideways with its muzzle. Immediately after the end of the third solvable trial, the experimenter repeated the procedure by blocking the container on the lid. The test phase lasted 60 s. The caregiver and the stranger were previously instructed to remain stationary and neutral, ignoring the cow for the entire duration of the unsolvable trial. At the end of the test, the experimenter came back to the test arena and opened the container, allowing the cow to eat.

### 2.3. Behavioral Assessment

A video camera was placed in front of the cow’s head, at a distance of about 3 m, set to record all elements of the experimental setting (i.e., people, apparatus, and testing cow) and the cow’s behaviors during the test. A trained researcher analyzed the behaviors (Table 1) with continuous sampling methods using the Solomon Coder^®^ beta 16.06.26 software (ELTE TTK, Budapest, Hungary). The inter-observer reliability was assessed by comparing the data to those obtained by a second independent blind coder on the 20% of the samples: the agreement between the two coders was very high for all behaviors (range from 97% to 99%). The duration (i.e., the time expressed in seconds) and the latency (i.e., the time in seconds from the start of the phase to the first occurrence of the behavior) of each behavior were collected. Solvable trials were not standardized in duration and were not included in the analysis, as they were performed only to properly trigger the violation of expectancy in the unsolvable trial.

### 2.4. Blood Sampling and Hormones Essay

Although sampling blood from cows may appear a non-invasive procedure, it remains a stressful event potentially affecting the behavioral outcomes during the test. Therefore, we avoided blood sampling before the test. On the other hand, multiple sampling after the test suffers the same problem: we could not exclude a hormonal response due to the sampling resulting in a bias on a second blood sampling. For these reasons, we have chosen to rely on only one sampling performed immediately (less than a minute) after the end of the test. Eight mL of blood were withdrawn from the coccygeal vein into plastic tubes and centrifuged at 4 °C and 1500× *g* for 15 min to obtain serum. The serum was divided into three aliquots, placed in plastic tubes, and stored at –20 °C until analysis. The average calculated over two dosages was used for the result. The serum concentration of oxytocin [51] and cortisol [52] was measured using competitive enzyme immunoassay (EIA) following the manufacturer’s instructions. For the Oxytocin ELISA kit, (Enzo Life Sciences, Lausen, Switzerland), the wavelength was set at 405 nm and the kit sensitivity ranged from 15.6 to 1000 pg/mL; for the Bovine Cortisol ELISA Kit (MyBioSource, Inc., San Diego, CA, USA), the detection range was from 0.049 up to 200 ng/mL and the wavelength was set at 450 nm.

### 2.5. Statistical Approach

The Kolmogorov-Smirnov test demonstrated that some of the parameters were not normally distributed, whereby we adopted a non-parametrical statistical approach. To test whether the cows preferred to interact before with the owner or the stranger we used a two-tailed binomial test with a test value of 0.5. The variables, cows’ age, cortisol levels, OXT levels, caregiver-directed behaviors, stranger-directed behaviors, and apparatus-directed behaviors were used for analysis by Spearman’s correlation, both for the duration and the latency. We have considered multiple comparisons adjusting significant *p* values according to Bonferroni correction. All statistical tests were performed by IBM SPSS statistic, version 26 (IBM Corp., Armonk, NY, USA).

## 3. Results

All cows solved the three solvable trials and were successfully enrolled in the test phase. The cows generally interacted with the apparatus as soon as the unsolvable trial began, with the exception of one subject that remained inactive for the whole test after passing the training phase. However, removing this cow from the dataset did not affect the results, therefore the analyses were based on the whole database. As a descriptive analysis, the duration of the apparatus-directed behaviors was (Mean ± SD) 40.59 ± 17.55 s (min = 0; max = 60.00); the duration of the caregiver-directed behaviors was 4.37 ± 6.47 s (min = 0, max = 31.60), the duration of the stranger-directed behaviors was 3.67 ± 4.80 s (min = 0, max = 19.80), OXT levels were 390.72 ± 113.55 pg\mL (min = 148.00; max = 576.00), and the cortisol were 13.79 ± 6.12 ng\mL (min = 6.90; max = 32.40).

**Figure 1:**
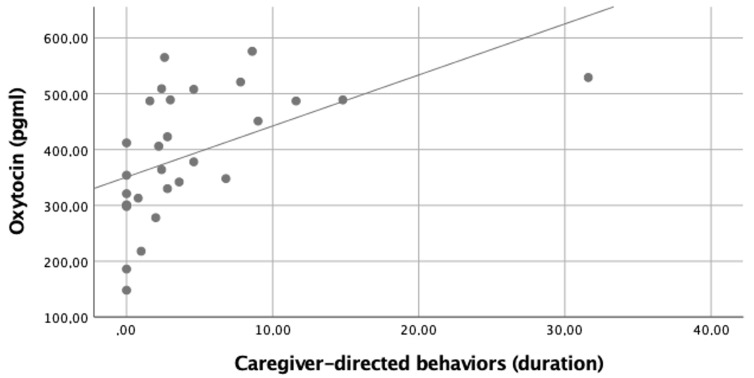
Simple scatter with fit line of serum oxytocin levels by the duration of the caregiver-directed behaviors. Spearman correlations between variables ρ = 0.699 (*p* < 0.001 with Bonferroni correction).

The test for comparing cow’ preferences demonstrated no differences between the stranger and the caregiver (*p* = 0.690). Correlation analysis revealed that the duration of the caregiver-directed behaviors was positively correlated with the OXT levels (Figure 1). No other significant correlation was found (Table 2).

**Figure 2:**
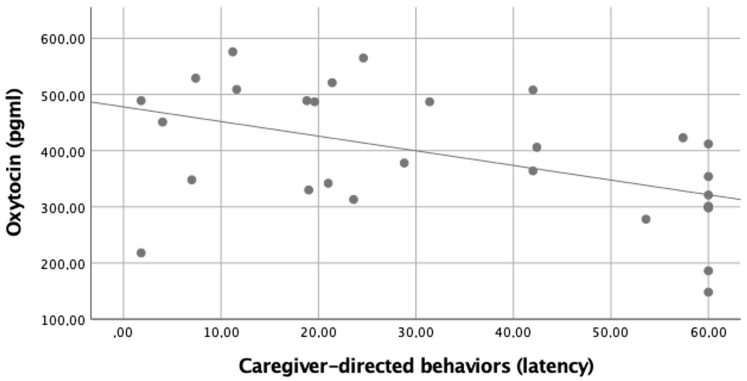
Simple scatter with fit line of serum oxytocin levels by the latency of the caregiver-directed behaviors. Spearman correlations between variables ρ = −0.490 (*p* = 0.042 with Bonferroni correction).

The latency of the apparatus-directed behaviors was 0.00 ± 2.07 s (min = 0; max = 60.00); the latency of the caregiver-directed behaviors was 1.80 ± 33.46 (min = 0, max = 31.60); and the latency of the stranger-directed behaviors was 0.80 ± 34.19 (min = 0, max = 19.80). The latency of the caregiver-directed behaviors was negatively correlated with the level of OXT (Figure 2). No other correlation was found (Table 3). ** *p* < 0.01

## 4. Discussion

Serum OXT levels were positively correlated with the duration and negatively correlated with the latency of the cows’ social interactions with the caregiver, thus supporting our prevision of a positive link between OXT and social behaviors in cows. Cows with higher OXT levels performed longer and faster social contact with the caregiver. The cows’ tendency to perform social contact with the caregiver was not correlated with that of the stranger, suggesting that, irrespectively of the familiarity, there is no general tendency in cows to make social contact with humans. Furthermore, the positive correlation with the caregiver did not result in a negative correlation with the stranger, as shown in some studies in humans [50] and dogs [35]. We found no correlation between cortisol and social behaviors. Actually, OXT in the blood occurs with a pick on average three minutes after stimulation [53,54,55], while cortisol release occurs on average with a pick 15 min after stimulation [54,55]. We sampled blood immediately after the end of the test (i.e., about 5 min from the beginning of the test), which may have allowed us to detect OXT responses, but not the cortisol response. Therefore, we cannot completely exclude a possible correlation between the two hormones and a possible relationship between the testing procedure and the stress levels in this species.

A correlation confirms a logical nexus between two variables, but does not provide information on a causal relationship. In other words, did higher levels of OXT increase the cows’ willingness to seek social contacts with the caregiver, or did the increased seeking of social contacts increase serum OXT levels in our test? We cannot address this concern, as we only collected one blood sample for each cow after the test. Previous studies have shown that several stimuli can drive the synthesis and release of OXT into the bloodstream [56,57] and some of these are just social stimuli as shown in human–human [7,58], human–dog [59,60,61], and human–lamb [14] studies. However, in all cited research, the increase of OXT levels in the serum was related to “feel good” activities through a gentle human-mediated interaction. In contrast, in the current study, people were passive and unresponsive to cows’ solicitation. This condition has been shown not to elicit the OXT release in dogs [17,19,62]. Furthermore, the release of OXT in the bloodstream seems to not be sensitive to social contact in cows, as a study failed to find any difference in the OXT levels of calves and adults before and after manipulation of social interaction [15]. Based on this literature, we can only suppose that the sampling procedure in our study reflects baseline OXT levels, but more studies are needed to address this issue.

Another concern is whether serum OXT could affect brain functioning. This peptide is mainly synthesized in the supraoptic and paraventricular hypothalamic nuclei and acts as a neuromodulator in many brain regions [63,64], or it is released into the bloodstream through nerve terminals from the posterior pituitary [65]. It has recently been established that circulating OXT can enter the brain via receptors for advanced glycation end products in endothelial cells, whereby acting on the brain [66]. Specific behavioral effects have been observed after transport from the plasma into the brain [67], and some studies have found a correlation between circulating OXT and behavioral outcomes [68,69]. For example, subcutaneous OXT injection restored maternal nurturing behavior in mutant mice for impaired social recognition [70] and facilitated social recognition in normal rats [71]. Therefore, both central and peripheral OXT may play a role in driving brain functioning and resulting behavioral responses, although it remains unclear whether and how peripheral and central OXT synergize [64]. This body of research lets us consider a possible causal effect of OXT on cows’ social behavior with humans. Our data, in fact, do not allow a definitive conclusion. Further studies are needed to demonstrate that social behaviors correlate with the basal serum OXT levels in cows and do not simply reflect the release of OXT from the pituitary as a consequence of the triggering situation.

Our results show that cows have no preferences for the owner or the stranger. A study using the IT demonstrated owner preference over the stranger in pet dogs by analyzing the latency of gazing behavior [49], but this finding has not been replicated in other studies [46,47,48]. No difference in the duration of the visual approach and social interactions was recorded in such studies. The IT failed to express a clear preference for human subjects according to familiarity probably because people were instructed to ignore animal solicitations. In dogs, specific training may increase owner salience in the IT, as in the agility [46] and water rescue dogs [47], but not in guide dogs for impaired visually people [48]. The cows in the current study were reared according to a semi-extensive system, which probably did not allow cows to develop social interactions towards the caregiver over the stranger, but it cannot be excluded that other breeding systems may lead to different results. In addition, it would be interesting to understand whether appropriate programs aimed at increasing sociability with humans could allow cows to develop a preference for a specific reference figure, as caused by some training processes in dogs [46,47]. The meaning of the social interaction of cows with people is not easy to interpret. Could cows be asking for help after the expectancy violation? Recent research applying the IT in dogs demonstrated that the gazing behavior toward people could not be considered a request for help [72]. To consider the cows’ social contacts as a request for help, we should have observed a positive correlation with the task and a faster interaction with humans after the expectancy violation, but this was not the case. Therefore, the social contacts observed in our study might simply reflect an alternative behavior towards salient stimuli after realizing that the feed was no longer reachable, as proposed for dogs [73,74].

## 5. Conclusions

Our study provides the first evidence in cows for a clear correlation between serum OXT levels and the willingness to engage in social contact with the caregiver and highlights that this effect was not affected by different stress responses. Overall, our results indicate that the mechanisms underlying OXT in the intraspecific social responses among cows [15,16] are mirrored in interspecific relationships with humans. The importance of the interaction between humans and farm animals for productivity as well as for animal welfare is accepted worldwide, but the possible implications are numerous and worth further investigation. Caregiver behavior towards cattle can generate animal responses that affect behavior, productivity, and health by primarily affecting the neuroendocrine system. Importantly, negative interactions can lead to physiological imbalances that can reduce production but also immune response, predisposing the animals to diseases [74]. Despite this, as observed, most of the literature on this topic reports studies in other species. Thus, further studies on this topic are strongly needed to identify the mechanisms underlying the best farm management in terms of interaction between humans and farm animals.

## Figures and Tables

**Table 1:** Ethogram in the impossible task.

Behavior	Description
Human-directed	Behaviors directed at the people (i.e., caregiver and stranger) aimed to gain physical contact with them (e.g., touch, push, licking, biting).
Apparatus-directed	Behaviors directed at the apparatus in an attempt to open it and reach food (e.g., touch, push, licking, biting, nibbling).
Other	Behaviors not included in previous descriptions (e.g., passivity).

**Table 2:** Spearman correlations between oxytocin, cortisol, and behavioral variables (duration). Asterisks indicate a significant correlation. Sample size: 29 cows.

		Cortisol	Oxytocin	Apparatus-Directed Behaviors	Caregiver-Directed Behaviors	Stranger-Directed Behaviors
Age	Coefficient	0.156	0.035	−0.235	−0.007	0.049
	*p* value	1	1	1	1	1
Cortisol	Coefficient		−0.271	−0.114	−0.032	0.148
	*p*-value		0.926	1	1	1
Oxytocin	Coefficient			−0.065	0.699	0.326
	*p*-value			0.738	<0.001 **	0.504
Apparatus-directed behaviors	Coefficient				−0.227	−0.348
	*p*-value				1	0.386
Caregiver-directed behaviors	Coefficient					0.257
	*p*-value					1

**Table 3:** Spearman correlations between oxytocin, cortisol, and behavioral variables (latency). Asterisk indicates a significant correlation. Sample size: 29 cows. * *p* < 0.05.

		Cortisol	Oxytocin	Apparatus-Directed Behaviors	Caregiver-Directed Behaviors	Stranger-Directed Behaviors
Age	Coefficient	0.156	0.035	0.079	0.195	0.068
	*p*-value	1	1	1	1	1
Cortisol	Coefficient		−0.271	0.045	−0.032	0.148
	*p*-value		0.926	1	1	1
Oxytocin	Coefficient			0.316	−0.490	−0.348
	*p*-value			0.568	0.042 *	0.387
Apparatus-directed behaviors	Coefficient				0.240	0.229
	*p*-value				1	1
Caregiver-directed behaviors	Coefficient					0.428
	*p*-value					0.124

## Data Availability

The datasets generated during and/or analyzed during the current study are available from the corresponding author on reasonable request.
